# How can we improve migrant health checks in UK primary care: ‘Health Catch-UP!’ a protocol for a participatory intervention development study

**DOI:** 10.1136/bmjopen-2025-106484

**Published:** 2025-11-29

**Authors:** Jessica Carter, Felicity Knights, Kathryn Mackey, Anna Deal, Eltayeb Hassan, Jose Trueba, Nimal Jayawardhena, Janet Alfred, Isra Al-Sharabi, Yusuf Ciftci, Nathaniel Aspray, Philippa Harris, Subash Jayakumar, Farah Seedat, Nuria Sanchez-Clemente, Rebecca Hall, Azeem Majeed, Tess Harris, Ana Requena Méndez, Dominik Zenner, Sarah Tonkin-Crine, Sally Hargreaves

**Affiliations:** 1 The Migrant Health Research Group, School of Health and Medical Sciences, City St George’s University of London, London, UK; 2 Wolfson Institute of Population Health, Queen Mary University of London, London, UK; 3 Migrant Health Community Research Network, London, UK; 4 London School of Hygiene & Tropical Medicine, London, UK; 5 Southwark Refugee Forum, London, UK; 6 Aymara, London, UK; 7 Refugee Council, London, UK; 8 RESPOND, University College London Hospitals NHS Foundation Trust, London, UK; 9 NHS Northwest London Integrated Care System, The Stonebridge Practice, Harness PCN South, London, UK; 10 Guy's and St Thomas’ NHS Foundation Trust, London, UK; 11 Primary Care and Public Health, Imperial College London, London, UK; 12 Population Health Research Institute, City St George’s University of London, London, UK; 13 Barcelona Institute for Global Health (IS Global Campus Clinic), Barcelona, Spain; 14 Centro de Investigación Biomédica en Red de Enfermedades Infecciosas, CIBERINFEC, ISCII—CIBER de Enfermedades Infecciosas, Instituto de Salud Carlos III, Madrid, Spain; 15 Queen Mary and Barts Health Tuberculosis Centre, Faculty of Medicine and Dentistry, Queen Mary University of London, London, UK; 16 Nuffield Department of Primary Care Health Science, University of Oxford, Oxford, UK

**Keywords:** Health, Primary Health Care, Preventive Health Services, INFECTIOUS DISEASES, Refugees, Cardiovascular Disease

## Abstract

**Abstract:**

**Introduction:**

Global migration has steadily risen, with 16% of the UK population born abroad. Migrants (defined here as foreign-born individuals) face unique health risks, including potential higher rates and delays in diagnosis of infectious and non-communicable diseases, compounded by significant barriers to healthcare. UK Public Health guidelines recommend screening at-risk migrants, but primary care often faces significant challenges in achieving this, exacerbating health disparities. The Health Catch-UP! tool was developed as a novel digital, multidisease screening and catch-up vaccination solution to support primary care to identify at-risk adult migrants and offer individualised care. The tool has been shown to be acceptable and feasible and to increase migrant health screening in previous studies, but to facilitate use in routine care requires the development of an implementation package. This protocol describes the development and optimisation of an implementation package for Health Catch-UP! following the person-based approach (PBA), a participatory intervention development methodology, and evaluates our use of this methodological approach for migrant participants.

**Methods and analysis:**

Through engagement with both migrants and primary healthcare professionals (approximately 80–100 participants) via participatory workshops, focus groups and think-aloud interviews, the study aims to cocreate a comprehensive Health Catch-UP! implementation package. This package will encompass healthcare professional support materials, patient resources and potential Health Catch-UP! care pathways (delivery models), developed through iterative refinement based on user feedback and behavioural theory. The study will involve three linked phases (1) planning: formation of an academic–community coalition and cocreation of guiding principles, logic model and intervention planning table, (2) intervention development: focus groups and participatory workshops to coproduce prototype implementation materials and (3) intervention optimisation: think-aloud interviews to iteratively refine the final implementation package. An embedded mixed-methods evaluation of how we used the PBA will allow shared learning from the use of this methodology within the migrant health context.

**Ethics and dissemination:**

Ethics approval granted by the St George’s University Research Ethics Committee (REC reference: 2024.0191). A community celebration event will be held to recognise contributions and to demonstrate impact.

**STRENGTHS AND LIMITATIONS OF THIS STUDY**

- Engagement and building of trust between the academic, clinical and migrant communities has been part of a wider continuous process prior to study conception.
- This study uses the formation of a community–academic coalition to ensure that migrant voices are central to the development process.
- The study employs the structured person-based approach to intervention development, combining behavioural theory and iterative end-user feedback.
- The embedded mixed-methods evaluation will capture how well our approach aligns with our coproduced engagement values.
- Recruitment through existing networks enables community engagement but may introduce selection bias towards more research-engaged individuals.

## Introduction and rationale

 There has been a continued rise in global migration in recent years due in part to the major political, environmental and economic events of the last two decades. Current estimates state that there are about 281 million international migrants in the world, equating to 3.6% of the global population.^1^ In the UK, the demographic landscape has shifted considerably, and it is now estimated that 16% of the population was born overseas, the majority of those outside of the European Union.^2^ Migrants (defined for this study as foreign-born individuals) contribute positively to their host society,^3^ but as was highlighted during the COVID pandemic, they face a disproportionate burden of poor health outcomes.^4 5^ Data show that some migrants are at higher risk of infections such as tuberculosis (TB), human immunodeficiency virus (HIV), hepatitis B/C and chronic parasitic infections such as Chagas Disease. These groups typically face delays to diagnosis and consequently poorer prognosis, as well as being at greater risk of underimmunisation and impacted by outbreaks of vaccine-preventable diseases.^4^ In addition, migrants from some groups have also been shown to be at increased risk of non-communicable diseases, such as diabetes and cardiovascular disease, dependent on the country of origin, country of destination and duration of residence.^6 7^ This picture is compounded by the existence of multilevel access barriers to national health services.^8 9^ The reduction of these health inequities for potentially vulnerable groups has been made a key government priority (National Health Service (NHS) Long-Term Plan), and primary care is well situated but underused in supporting prevention and early diagnosis through proactive migrant health screening for key conditions.^10 11^

A systematic review of infectious disease screening interventions for migrants in the European Union (EU) found that European countries have adopted a variety of approaches to screening migrants.^12^ These, however, are often limited in scope to single diseases and a narrow subset of migrants and have low coverage.^12^ The UK Health Security Agency’s (UKHSA) migrant health guide supports a more holistic approach to migrant health, suggesting increased screening for multiple diseases (both communicable and non-communicable).^13^ This aligns with national and global targets to eliminate key infections such as viral hepatitis and TB as public health problems and promote early diagnosis of chronic conditions among inclusion health groups.^14^,^17^ The challenge facing primary care is developing and successfully implementing screening interventions to deliver this guidance, which meet both the needs of a heterogeneous migrant population and a diverse and increasingly overburdened primary healthcare landscape. This challenge is reflected in current UK screening interventions for at-risk migrants, such as the national latent TB infection screening programme in primary care, which reports low uptake despite clear potential NHS health and cost benefits.^11 18^ Our UK-based qualitative research study identified key barriers in primary care to provide screening and catch-up vaccination to at-risk migrants at staff-level (inconsistency in delivery, lack of disease knowledge and awareness of screening/treatment pathways), system-level (challenges identifying at-risk patients, migrant healthcare not prioritised, workload) and patient level (lack of patient awareness/engagement with screening, digital exclusion compounded by pandemic).^11 18 19^ Recent work on integrated multidisease migrant health screening in primary care (screening for more than one condition at one time point) suggests it could be an effective strategy for migrant groups and overcome some of these barriers. For example, the IS-MiHealth tool trial in Spain and the community-based testing of migrants for infectious diseases (COMBAT-ID) study in the UK show the potential for better uptake, feasibility and acceptability of primary care based multiscreening interventions.^20^,^24^

**Figure 1:**
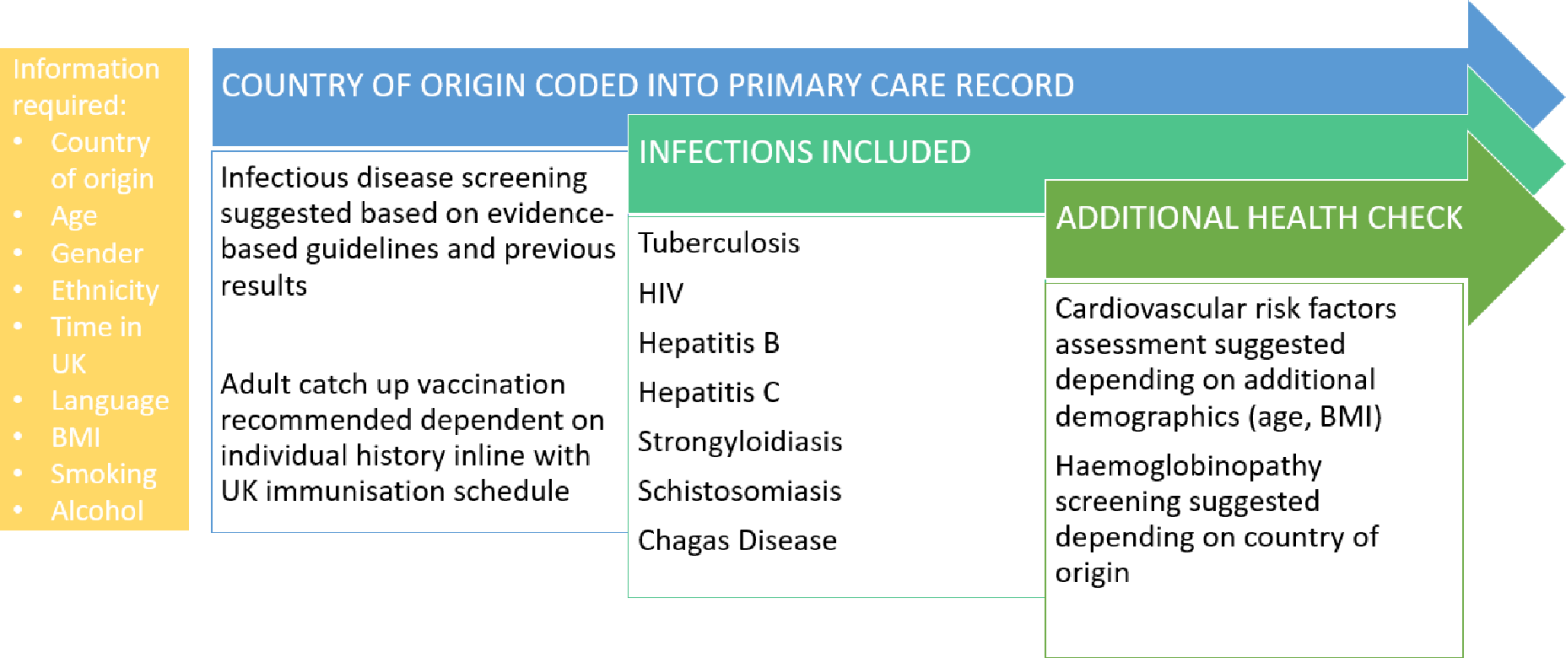
Summary of the Health Catch-UP! clinical decision support tool. (BMI, body mass index).

In response to these challenges, we developed the Health Catch-UP! tool through collaborative engagement with stakeholders, including migrants, digital-health specialists and healthcare professionals.^11 25^ This is a two-step digital clinical decision support tool that sits within the GP software EMIS (Egton Medical Information System) and supports healthcare professionals in identifying at-risk migrants through first, the coding of key demographics and then producing an appropriate individualised screening prompt based on national guidelines (National Institute for Health and Care Excellence and UKHSA).^13 25^ The Health Catch-UP! tool is outlined in figure 1 below.

Initial evaluation of the Health Catch-UP! tool demonstrated that use of the tool improved migrant demographic data collection, resulted in consistent comprehensive migrant screening offered by primary healthcare professional (PHCP) and had high screening uptake.^13 25^ The approach was reported as highly acceptable to end-users and was feasible in this setting, with PHCPs and migrants enthusiastic about the support it provided in clinical decision making and facilitating delivery of preventative healthcare.^25^ There were, however, implementation challenges. These included questions around how to engage PHCP with the intervention, understanding most appropriate Health Catch-UP! care pathways/delivery models, integration with new digital communication methods (eg, interactive text messaging) and how to improve engagement with marginalised migrant populations, taking into consideration barriers of trust, stigma, language and the diverse experience and expectation of healthcare provision.^25^ PHCPs and migrants alike highlighted the need for patient and PHCP support materials, including training, videos and infographics to improve knowledge and awareness of the tool and the diseases screened for, alongside clear delivery pathways for PHCPs to follow and flexible strategies for communication of the Health Catch-UP! offer.^25^ Similar implementation barriers were found during the IS-MiHealth trial, which highlighted the particular importance of communicating the eligibility and offer for screening in a culturally appropriate way, taking account of language, literacy, gender and background.^26^

To realise the benefits of the Health Catch-UP! approach; ensuring PHCPs use the tool, and migrant patients engage with it, we have identified that collaborative development involving the target population (migrants and PHCPs) of a multifaceted implementation package is required.^27^ We therefore seek to conduct an innovative participatory intervention development study using the person-based approach (PBA) to ensure it is acceptable and feasible for adult migrant populations and PHCPs.^28 29^ The PBA is a systematic methodology for developing behavioural interventions that combines evidence and theory with participatory qualitative research involving target users throughout the development process.^28^ We recognise that participation and participatory are often overused terms and have expanded on our interpretation and use in this manuscript in Box 1 below. Participatory methodologies such as the PBA are recognised as a best practice approach to collaboratively address social determinants of health and reduce inequalities, essential when working with potentially vulnerable groups such as those with lived experience of migration.^29 30^ Such methods are more effective than traditional research approaches at breaking down barriers to involvement reported by these groups through the emphasis on the sharing of power between academics and participants.^2729^,^31^

Box 1 **A note on the challenges of the definition of participation/participatory research**

We recognise that ‘participatory research’ is often used broadly to describe various forms of community engagement. For this study, we distinguish between participatory and consultative approaches. While our methods include some traditional qualitative techniques (workshops, interviews and focus groups), what makes our application of the person-based approach in this research study participatory is a shift in power dynamics.

We define our participatory approach as research where migrant community members and healthcare professionals are not just consulted or involved as data sources, but actively co-own the research process through shared decision-making in intervention design; collaborative analysis and interpretation of findings; joint problem-solving when challenges arise; and collective ownership of research outputs and dissemination

The academic–practice–community coalition exemplifies this approach, with community members holding equal authority in research decisions alongside academic and clinical partners. This contrasts with research where participants are asked predetermined research questions but do not shape the fundamental direction or interpretation of the work.

The proposed research represents an important, timely and under-researched area. The barriers to deliver migrant healthcare are well documented, but there is limited research in the codevelopment of solutions, or in using the PBA with migrant communities.^29 32^ This study has the potential to inform other screening initiatives to include health groups who typically experience multiple overlapping risk factors for poor health.^33^ This aligns with current national government and international targets for disease elimination and tackling health inequities.^10^

### Preparatory community engagement

**Figure 2:**
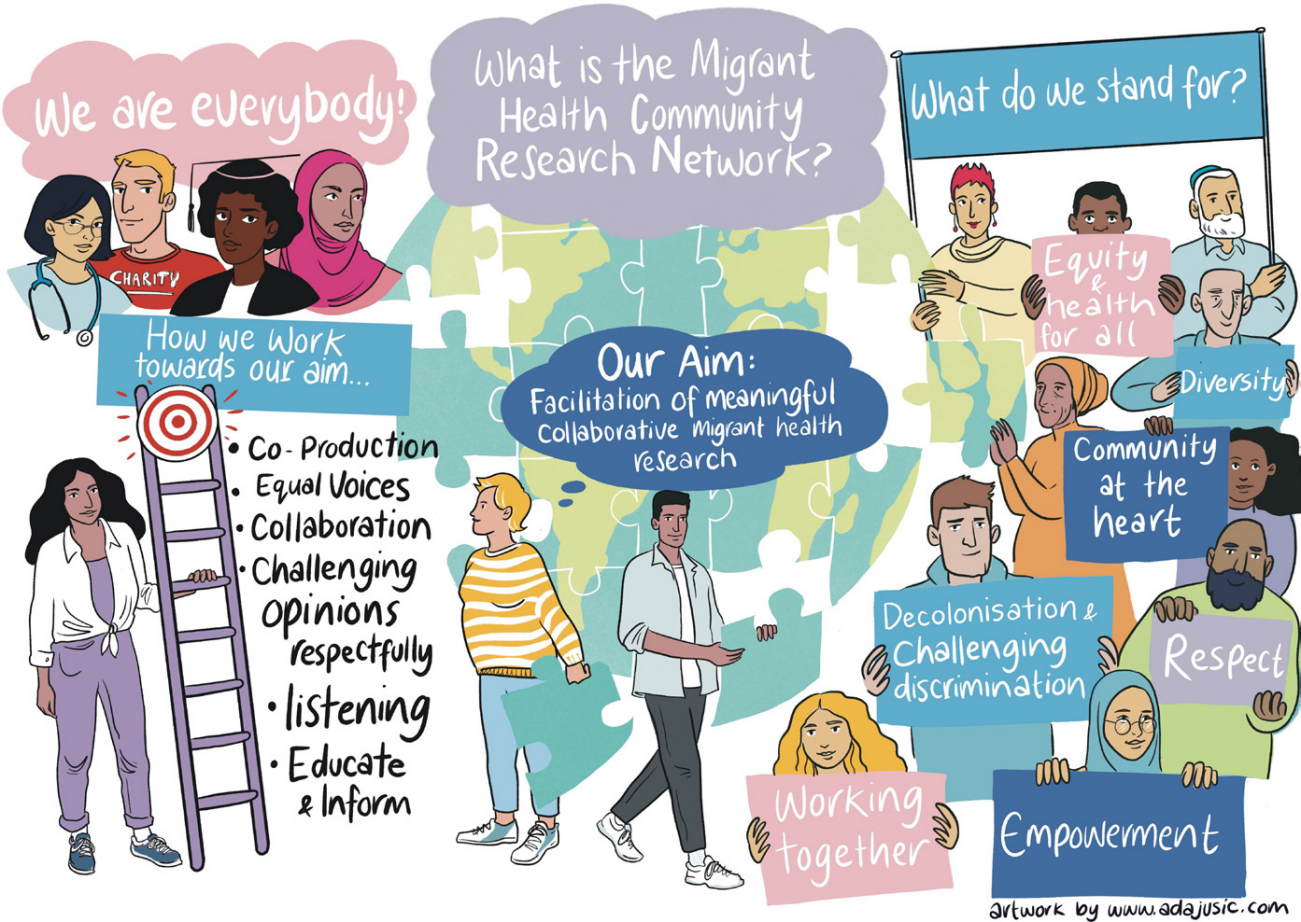
Visual representation of the migrant health community research network (artwork Ada Jusic).

Our migrant health research group at City St George’s, University of London, where this study is based, has consistently worked in collaboration with communities with lived experience of migration. Based on this experience and listening to the voices of these communities, we have established the Migrant Health Community Research Network (MHCRN) (https://www.migranthealthnetwork.org/), visually represented below in figure 2. The shared aim of the network is to work sustainably in partnership to cocreate community-centred migrant health research from inception to delivery in a proactive effort to move away from the current academic ‘norm’ of project-by-project community engagement, which propagates the potentially tokenistic nature of academic–community partnerships.^27 30 31 34^ The iterative development of the Health Catch-UP! intervention outlined in this protocol is embedded in the MHCRN ethos to challenge the barriers faced by migrants in accessing equitable primary healthcare.

Preliminary work for this study included two in-person workshops with the MHCRN in July 2023 and January 2024, led by JC, FK and YC. At these workshops, the Health Catch-UP! intervention was presented to 50 members of the MHCRN from diverse backgrounds (representation from 28 different community groups). An interactive discussion then took place in the form of an ideas cafe, used to gauge interest and explore engagement in the further intervention development.^35^ Interest in Health Catch-UP! from the MHCRN was high. Participants were invited to share their views on the format of an implementation package for Health Catch-UP!, including patient support materials and potential care pathways/delivery models that would be acceptable and feasible for the migrant population in the UK primary healthcare setting. These discussions are summarised in table 1 below.

**Table 1:** Preliminary outline of the Health Catch-UP! implementation package

Implementation package component	Key features	Reason
Patient support materials: Use of mixed media, including videos, leaflets and text messages, to prepare/‘give a head’s up’ that an offer of specific migrant health screening is available	Diverse patient population Diverse range of PHCPs Personal narratives, both positive and negative Easy to share format (eg, video link) Multiple languages	Introduce the concept of preventative healthcare Engage the target population through recognition of potential risk Sharing of knowledge and awareness of Health Catch-UP! offer through a variety of channels
Potential care pathways/delivery models: Use of community outreach from National Health Service PHCPs to where the patients are	Community-based information sharing workshops Involvement of community leaders Potential training of community ambassadors/patient champions	Increase trust and relationship building between the community and the PHCP Reduce the power differential between PHCP and the community Sharing of knowledge and awareness of Health Catch-UP! offer through a variety of channels
PHCP support materials: Culturally competent training materials for PHCP informed by the community with lived migration experience	Outline reasons migrant screening is required, including health consequences and equitable healthcare Case studies of good and bad migrant health screening interventions Lived experience stories from diverse groups ‘How to’ guide to deliver culturally competent risk assessment and screening offers	Increase awareness, engagement and motivation of PHCPs with migrant health Reduce perpetuation of stigma associated with screening for certain conditions Increase trust and relationship building between the community and PHCP Increase PHCPs’ confidence with migrant health

PHCP, primary healthcare professional.

## Methods and analysis

### Study coalition formation and positionality of clinicians and academics

Guided by principles of participatory research, five members of the MHCRN with lived experience of migration who had expressed interest in further involvement in the Health Catch-UP! project were approached to create an academic–practice–community coalition alongside three PHCPs with an interest in migrant health and the Migrant Health Research Group academic team led by JC and FK.^31 36^ The formation of this Health Catch-UP! coalition with balanced representation ensures multiple perspectives inform the development, analysis and dissemination process while maintaining strong connections to the academic, NHS primary care and community contexts of our work.^31^

As individual members of the coalition, we simultaneously occupy spaces of advantage and disadvantage. The healthcare professionals and academics in our coalition may hold institutional power, medical expertise and research authority, yet depend on community members’ lived experiences and cultural knowledge. Community members may experience marginalisation based on migrant status and cultural background, but possess crucial insights about their communities’ needs and effective engagement strategies. The coalition’s academic and clinical professional statuses may carry less weight when navigating community networks, while community members’ experiential knowledge becomes essential for project success. These positions challenge traditional hierarchies, and we aim to remain cognisant and reflective of our roles throughout this project.^37^

At initial coalition meetings (four online hour-long meetings held November–February 2024), the group discussed relevant experience, project expectations, coalition values, timelines and budget. The importance of inclusivity of all the participatory workshops was highlighted, and suggestions to allow for this included the hosting of these in community settings, the use of interpreters and the accommodation of multiple languages to ensure a diverse range of views captured.^29^

Concurrently, the researchers continued to engage the broader MHCRN through a 1-hour online meeting to coproduce a set of core commitments against which we will evaluate our participatory work. These commitments were informed by both the UK Standards for public engagement and the previously agreed MHCRN values. The commitments will indicate if and how we as a network are acting in accordance with our fundamental values. As such, these commitments form the basis of the evaluation, we would have ‘succeeded’ in this participatory research study (we define ‘success’ as meaningful participant engagement in the research process) if we meet these core commitments.^38^ The agreed commitments are listed in box 2 below.

Box 2 **Core commitments**

Core commitments to operationalise our participatory research values

1. Creation of a safe space for participants to share
2. Value all contributions from all participants
3. Empowerment of participants and support to share
4. Value the diversity of participants and contributions

### The person-based approach

The PBA is a method for developing health interventions that combines behavioural theory (in our case, the COM-B model for behaviour change, capability, opportunity and motivation), empirical evidence from the literature and qualitative research.^28 39^ It emphasises understanding the experiences, needs and contexts of target users (migrants and PHCPs) to ensure the intervention is acceptable, engaging and feasible.^28 40^ This study will employ the principles of the PBA to iteratively develop and optimise an implementation package for Health Catch-UP!.^28 41^ The PBA was chosen because it is a systematic approach to develop interventions suitable for real-world settings like primary care.^28 42^

As outlined by the PBA, the study will take the format of three phases: intervention planning, intervention prototype development and intervention optimisation^43^ and involves five main participant activities:

1. Coalition co-production meetings and ongoing engagement with the MHCRN.
2. Participatory workshops with migrants.
3. Participatory workshops and focus groups with PHCPs.
4. Qualitative think-aloud interviews with migrants and PHCPs.
5. Embedded mixed-methods evaluation of the PBA participatory approach.

**Figure 3:**
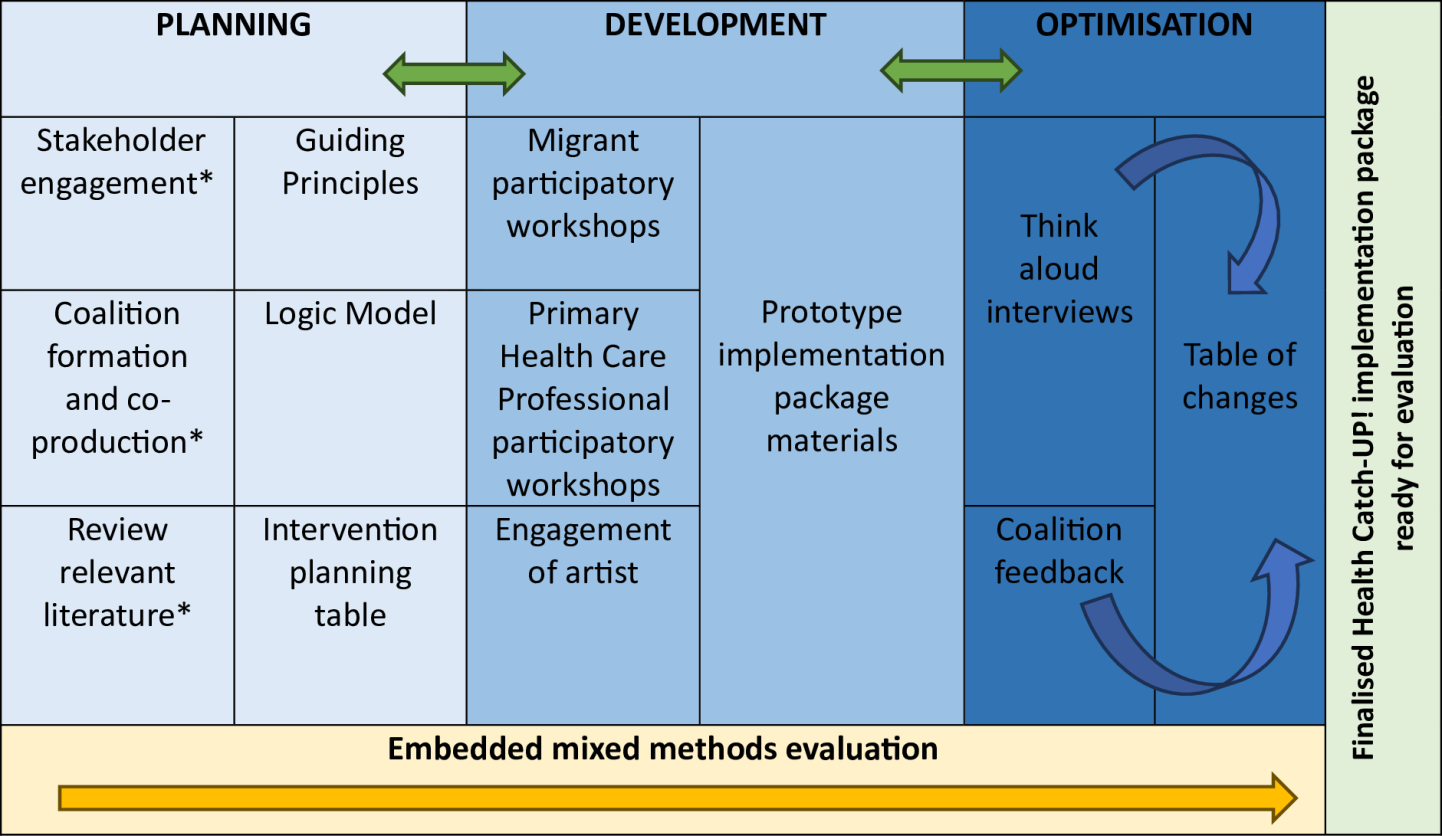
Outline of the Health Catch-UP! implementation package development study (*preparatory work already done).

The study design is summarised in figure 3 below with an evaluation component embedded across all phases. It is set out in three interconnected phases; however, as this is an iterative process, it is envisaged that there will be movement back and forth between phases to allow for continuous learning and intervention optimisation.^28 41^ This protocol reports on the decisions made regarding the study design to date, and the full process will be written up at the end of the study.

### Setting and population

This study will be carried out in London, a highly diverse city with over 40% of its residents born abroad and with over 300 languages spoken, but with the option for online participation to allow for representation across England.^2 44^ The study will be conducted with adult migrants (>18 years) defined as foreign-born, and with clinical PHCPs (general practitioners, nurses, healthcare assistants) currently working in the NHS primary care setting in England. Specific inclusion and exclusion criteria are described in table 2.

**Table 2:** Inclusion and exclusion criteria

Target population	Inclusion criteria	Exclusion criteria
Migrants	Born outside of the UK Aged 18 or above Currently residing in the UK Willing and able to give consent	Not a migrant Below the age of 18 Temporarily in the UK (eg, holiday/visiting friends and relatives) Lack capacity to consent as determined by the mental capacity act framework
Clinical PHCPs	Aged 18 or above Clinical PHCPs, including GPs, nurses and HCAs. Currently working in UK primary care Willing and able to give informed consent	Not a clinical NHS PHCP Below the age of 18 Individuals who lack the capacity to consent as determined by mental capacity act framework

GPs, general practitioners; HCAs, healthcare assistants; NHS, National Health Service; PHCP, primary healthcare professional.

### Recruitment

Purposive and snowball sampling approaches to recruitment will be used.^45 46^ Migrant participants will be identified and recruited from the MHCRN (described above) and its constituent groups. PHCPs will be recruited through our existing professional networks, social media posts in relevant groups and snowball sampling from initial participants. The research will be conducted through a combination of in-person workshops at community centres and university meeting rooms, remotely via telephone/MS Teams or within a private room of the individual’s choosing. NHS sites will not be used for recruitment or any other part of the study.

### Data collection and activities

The study data and collection methods are outlined below and summarised in table 3. Due to cultural preferences, following discussion with the coalition and to avoid digital exclusion, migrant participant data will be collected face-to-face unless requested otherwise. PHCP data will be collected for the most part online to allow flexibility for participation. Participant information sheets will be distributed in advance of workshops, and interviews with participants will be given the chance to ask questions. Written informed consent will be obtained prior to starting workshops or interviews. Workshops and interviews will be facilitated by JC/FK/KM, the broader Migrant Health Research Group and members of the coalition, with interpreters depending on participant preference. Workshops will be guided by a predetermined agenda with specific prompts, group activities and questions. Think-aloud interviews will be collected through a pilot-tested topic guide to gain feedback on prototype intervention materials. All workshops and interviews will be audio recorded with Dictaphones or, if online, via MS Teams recording function and then translated where required and transcribed. Other data collected will include written data/notes/mapping activities and sketches resulting from various activities and written comments generated by the online workshops. The academic team will keep reflective diaries and meet regularly to debrief, discuss data and adapt the approach as necessary. All the planned data collection and activities are summarised in table 3, which are then expanded below.

**Table 3:** Summary of all study activities

Phase	Activity	Population	Data generated	Data collection methods
**Planning**	Coalition coproduction meetings (n∼10)	Academic–clinical/community coalition	Information about sociocultural context, customs and preferences Beliefs and lived experience of receiving and delivering preventative healthcare and catch-up vaccinations Suggestions for engagement approaches, interventions and implementation strategies	Online meetings Reflective diaries Field notes Emails Written feedback Guiding principles Logic model Intervention planning table
**Development**	In-person codesign workshops with migrants (n∼2)	Migrant community members (n∼30)	Beliefs and experiences related to preventative healthcare screening and catch-up vaccinations What makes good migrant health screening and catch-up vaccinations Codevelopment and iteration of intervention prototypes Suggestions for implementation Sociodemographic and primary care organisation information	Participatory workshops Post-it notes Interactive posters Live feedback sessions Field notes Demographics questionnaire
**Development**	Online focus groups/codesign workshops with PHCPs	Clinical PHCPs (n∼30)	Relationship of primary care with the migrant patient population and the role played in screening and catch-up vaccinations What makes good migrant health screening and catch-up vaccinations Codevelopment and iteration of intervention prototypes Suggestions for implementation Sociodemographic information	Participatory workshops Interactive chat/voting Field notes Demographics questionnaire
**Optimisation**	Qualitative think-aloud interviews with migrants and PHCPs	Migrant community members (n∼20) Clinical PHCPs (n∼20)	Feedback and iteration of intervention prototypes Implementation challenges in real-world context Sociodemographic information	Think-aloud interviews Table of changes Demographics questionnaire
**Concurrent evaluation**	Embedded mixed-methods evaluation of the PBA participatory approach	All populations plus coalition	Feedback on involvement in the participatory process Success at aligning with core study commitments Sociodemographic information	Reflective diaries Observation field notes Feedback questionnaires Postevent interviews/focus groups Demographics questionnaire

PBA, person-based approach; PHCPs, primary healthcare professionals.

### Phase one: intervention planning

The first phase focuses on the foundations for our intervention planning, beginning with the collaborative development of our *intervention guiding principles, logic model* *and an intervention development table*.^43^ This work builds on our and others’ reviews of the literature, our previous qualitative studies and the Health Catch-UP! study while incorporating input from our academic–practice–community coalition and MHCRN workshops to outline the core intervention features and the behavioural mechanisms targeted by the Health Catch-UP! implementation package intervention.^11 12 25 47^

#### Guiding principles

To facilitate the success of this project as a group, we will develop our Health Catch-UP! ‘guiding principles’. These are the overarching principles, based on what is already known about our users through previous research and PPI, outlining what is needed to make the Health Catch-UP! implementation materials acceptable, feasible and engaging for the target users (migrants and PHCPs) and help keep the development focused on what will be especially appealing and specifically useful to the intended Health Catch-UP! users. Development of our guiding principles will be an iterative process through consensus-building discussions with the coalition, and they will be revisited and refined throughout the project.

#### A logic model

A logic model (our theory of change) will be produced based on our review of the literature, our previous qualitative and feasibility studies and with input from the coalition to outline the behavioural mechanisms being targeted overall by the intervention (Health Catch UP!) and each of the core implementation material groups (patient support materials, PHCP materials, delivery models). To show how they should lead to positive behaviour change (appropriate use of, and engagement with, Health Catch-UP! to deliver screening and vaccination). Coalition members will actively contribute to logic model development through structured activities such as problem mapping, outcome prioritisation and mechanism identification, with decisions made collectively rather than by researchers alone.

#### Intervention planning table

To facilitate the iterative development of these materials, we will produce an intervention planning table for this project with the aim of bringing together all the available evidence about exactly what elements are needed in the intervention (Health Catch-UP! implementation materials) and why. The intervention planning table will systematically record key design decisions, evidence sources and rationales in a structured format, allowing transparent tracking of how different evidence types inform specific intervention components. This will draw on behavioural theory (the COM-B model), our previous and other published research, and the expertise of the community-academic coalition. This table will help by capturing key pieces of information and early decisions in one place so that it is easy for the whole team to see and discuss the evolving picture. An example table can be seen in the online supplemental file 1.

### Phase two: intervention development

The second phase encompasses intervention development in the format of implementation material prototypes. Two participatory workshops will be held with diverse migrant community groups in person in well-known and established community settings in Southwark, with around 15–20 participants at each workshop. The workshops will be participatory in nature because participants will be supported to make collective decisions about content and share authority in determining implementation approaches, rather than simply providing opinions on predetermined options. These sessions will be cofacilitated by coalition members and conducted in multiple languages (3–4) with interpretation support from the community to ensure accessibility and meaningful engagement from our diverse participant population. The workshops will introduce the Health Catch-UP! intervention, capture beliefs and lived experiences of receiving preventative healthcare, what participants feel good migrant health screening should look like, suggested models of delivery for Health Catch-UP! and what engaging patient support materials would look like. Approximately 3–5 PHCP workshops will be conducted online, with between 5 and 10 participants expected to last between 1 and 2 hours. Participants will be introduced to the Health Catch-UP! tool and encouraged to share their experiences of providing preventative healthcare screening to migrant populations. Workshops will be designed as highly interactive sessions using small group work, collective prioritisation exercises and creative activities. There will be focused activities exploring implementation models for Health Catch-UP!, creation of potential implementation package components and generation of real-world implementation considerations. JC and FK will summarise data collected during these workshops and work with artists experienced in generating health-related communication materials, to generate prototype intervention materials for phase three.

### Phase three: intervention optimisation

Phase three will complement the participatory workshops using think-aloud interviews to look at specific details in the content of the prototype implementation materials. Think-aloud interviews are a type of qualitative interview that allows the research team to ask the end users to use and engage with the prototypes and say out loud the thoughts that come to mind as they work through them.^43 48^ Interviews will be used to identify areas of the materials that might require modification to maximise engagement, to check that information is coming across as intended, and to see how individuals navigate the materials.

It is envisaged that once the first draft of the implementation materials has been produced, we will conduct a first round of 3–4 think-aloud interviews per user group (migrants and PHCPs) to elicit feedback and make changes. A second round of interviews will then take place again with 3–4 participants per user group, with further changes in response to their feedback. It is envisaged that we will require between 15 and 20 interviews and therefore 3–4 rounds of interviews per group (migrants and PHCPs). The final drafts of the materials will also be sent to the workshop participants and discussed with the coalition for any feedback comments prior to finalisation. Depending on the type and number of implementation materials produced, interviews are predicted to last between 30 and 60 min. Interview participants should not have taken part in the workshops.

### Embedded evaluation

A contemporaneous embedded evaluation of the participatory research approach used in this study will be conducted to understand how well this study was able to achieve its objectives using the PBA and participatory research methods, enabling iterative learning of the study team and shared learning for wider academic audiences. The evaluation will focus on the following three questions:

1. To what extent did migrant participants feel meaningfully involved and like partners in the intervention development?
2. What was the experience of the migrant participants of being involved in this participatory study, and how could it be improved?
3. To what extent did this study align with the co-produced commitments and core values?

The evaluation will use researcher field notes and reflective diaries, participant self-reported experience questionnaires, workshop observations and one-on-one interview/focus group debriefs with facilitators. This triangulation of multiple data sources collected at different time points throughout the study will facilitate the capture of participants’ and facilitators’ experiences and perceptions during the participatory process. The evaluation will be overseen by a member of the research team not involved with the wider project (KM) and therefore acting as an external assessor to reduce bias. KM started this process through consultations with the research team and MHCRN, where we agreed ‘what success looks like’ in this study as outlined above. This will guide the evaluation and development of the questionnaire and debrief prompts, which will be iteratively created and piloted. The questionnaire and prompts, as well as the fieldnotes, KM’s observation notes and reflexive diaries, will explore representation, understanding of the research process and methods used, as well as levels of satisfaction regarding involvement and barriers and facilitators to meaningful involvement.

All those taking part in the migrant workshops and interviews are eligible and will be approached to take part in the evaluation component of the study, on an opt-out basis, which will form an optional part of the participant information sheet and consent form. We aim for at least half of the participants to provide some feedback. After completing the workshops or think-aloud interviews, participants who had consented to be part of the evaluation will be directed to the survey platform (Microsoft Forms) via email or via a QR code or paper version made available on the day. They will also be invited to take part in a short focus group or one-on-one interview debrief, either in person after the workshop/interview or over MS Teams, depending on preference.

### Data analysis

Demographic data will be collated and analysed in STATA to give descriptive statistics of demographic variables of participants. As the purpose of the participatory workshops is to codevelop intervention materials and prototypes, data from the workshops will primarily be analysed collaboratively, in real-time, during the workshops. The summary notes and audio recordings from the workshops and photographs of any visual data generated (eg, post-it notes, mind maps, photographs, drawings, infographics, field notes, etc) will be transcribed and subsequently imported into NVivo software for data management and further analysis with support from the coalition. This will contribute to the iterative development of our guiding principles, logic model and intervention planning table. Summaries of the workshop data will inform the artist collaborators’ production of prototype materials.

Think-aloud interview data will be audio recorded, transcribed verbatim, translated where required and imported into NVivo for management. Data will be analysed according to the PBA. JC and FK will use the interview data and fieldnotes to draw out key positive and negative comments about specific elements/features/sections of the intervention materials. These will be recorded in a table of changes, which will be used to systematically bring together all the feedback on experiences of the draft materials obtained from users.^28 43 49^ (see online supplemental file 1). Negative comments will support the team to identify what might need changing about the materials, taking into consideration whether changes are likely to impact behaviour change (engagement with Health Catch-UP!). The inclusion of positive comments will support discussion about whether to change elements that are liked by some participants but not all. This table of changes will be discussed with the coalition to find solutions to problems with the materials. To support decision-making, we will use the coding framework suggested by the PBA (see online supplemental file 1) to help decide how and why any proposed changes are important. In order to prioritise which changes to make (which are essential and achievable within our resources), the MoSCoW framework (Must have, Should have, Could have, Would have) will be applied.^43^ This table will be used after each round of interviews to allow for a transparent and chronological record of the iterative development of the materials, and the basis on which each decision was made.

Qualitative data from evaluation (including field notes, reflective diaries and interview and focus group debriefs) will be imported into NVivo software for data management and analysis against the study’s core values. The project quantitative evaluation questionnaire data will be collated and analysed using a statistical software package (STATA) to produce descriptive statistics. Formal coding and qualitative analysis of the open-text comments will be performed using NVivo.

### Schedule

The planned duration of the study is 12 months, starting from December 2024 and ending in December 2025.

### Support for partners

Study partners from Southwark Refugee Forum and MHCRN, as well as participants in workshops and interviews, will be financially compensated for their time and effort in line with National Institute for Health and Care Research INVOLVE guidelines.^50^

### Patient and public involvement

Patient and public involvement is inherently embedded throughout this study’s participatory design approach. The design of the study was informed following a world café event at the MHCRN day in July 2024, where over 50 members participated and contributed their views. The community–academic coalition with 5 members of migrant communities will oversee this project as described above.figure 3

### Ethics and dissemination

Ethics approval granted by the St George’s University Research Ethics Committee (REC reference: 2024.0191). A celebration event for participants and the local community will be organised at the end of the study to show the impact and recognise diverse contributions. The study findings will be disseminated at local, national and international levels, including through conferences, policy, stakeholder and voluntary/community sector meetings, peer-reviewed journals, a PhD thesis and multimedia outputs (eg, video clips and on social media). Preparation and recommendations for a future larger-scale study and testing of prototyped interventions will be made.

## Supplementary material

10.1136/bmjopen-2025-106484 online supplemental file 1
