# Supplementary material for: How can we improve migrant health checks in UK primary care: ‘Health Catch-UP!’ a protocol for a participatory intervention development study
Source: BMJ Open. 2025 Nov 29;15(11):e106484. doi: 10.1136/bmjopen-2025-106484 (PMC12666209; doi:10.1136/bmjopen-2025-106484)
Supplement: online supplemental file 1 [file bmjopen-15-11-s001.docx]

**Supplementary Files**

**Think Aloud Topic guides**

1. **INTRODUCTION**

During the Interview

- Start with a warm welcome and explain this isn't a test of the participant
- Demonstrate the process briefly ("Here's how I might think aloud...")
- Use simple instructions like "Please tell me everything you're thinking as you look at this"
- Stay neutral - avoid leading questions or showing approval/disapproval
- If participants fall silent, use gentle prompts like "What are you thinking now?" or "What's going through your mind?"
- Listen more than you speak
- Make notes of non-verbal cues (frowning, pausing, smiling)

*Introduction script guide*

- This is a study to develop an intervention to help GPs deliver screening and vaccination to migrants in the UK
- I have a copy of the information leaflet here that you will have received from the research team. This explains that we are trying to find out what people think of the intervention materials that have been designed for Health Catch UP! to try and make them as useful and easy to use as we can. I will be showing you a selection of materials and asking you to tell me about what you think of them. This shouldn’t last more than an hour, and you will get a £25 voucher at the end to thank you.
- If you want to withdraw at any time and not take part anymore, please just let me know.
- If you are happy, we will record the interview, so we can listen again to what is being said. Everything we talk about here will be confidential. We will take care to make sure that all the information you share with us is kept safely and securely.
- Is there anything you would like to ask me at the moment?
- OK, so I will tell you a little more about what we will be doing today. We’re interested in your views of the first version of the Health Catch-UP! intervention materials. All you have to do is use the materials as you would if I was not here and say your thoughts out loud.
- To help you think aloud you may find it useful to read aloud or tell me what you are looking at and why. You may find at times I will say aloud what you have picked up or what page you are looking at – this is just so when we listen to your views again we know what page you are talking about.
- This is not a test and you are not being judged. There are no right or wrong answers, so please say any thoughts which spring to mind, even if you think they might not be important. We just want you to say out loud any thoughts which are running through your mind.
- Please do feel free to say any negative thoughts you may have about the intervention materials as these will be really useful in helping us to improve it so please do be honest. Your views about the intervention are really important so the more you can tell us the better.
- It’s also important you understand that the intervention materials are still in development and not fully ready to use.
- I won’t be able to answer your questions as we go through, but I can answer anything at the end. So if you ask me a question while you’re looking at the materials, I will probably say that we can talk about it at the end.
- After you have finished looking at the materials, I would like to have a chat with you about your overall views of the intervention.
- We can take a break at any time you like, please just let know and I can pause the recording. We can also stop the interview at any time if you want to.
- Ok so if you are happy, please can you complete this consent form?
- Do you have any questions?
- Are you happy if I start recording now?

1. **THINK ALOUD AND RESEARCHER PROMPTS FOR EACH MATERIAL**

- What are your first impressions?
- What are you thinking now?
- What do you think about [this visual, this information, text]?
- Can you tell me a bit more about why you think that?
- What is it you like about that?
- That’s really interesting./…..

Helpful Prompts

- "What do you think about this section?"
- "What stands out to you here?"
- "What are you noticing as you read this?"
- "What's your reaction to this part?"
- "What questions do you have at this point?"

1. **POST THINK ALOUD QUESTIONS**

- Overall, what do you think about the intervention?
- Can you tell me about anything you thought was particularly good about the intervention materials?
- Can you tell me anything about the intervention materials that you were less keen on?
- What do you think should be changed?
- What do you think it would be like using the intervention materials in real life?

1. **THANK YOU**

Ensure completed socio-demographic questionnaire

Voucher

Goodbye

Common Challenges and Solutions

- Participant goes quiet: Gently remind them to keep sharing thoughts
- Participant asks questions: Redirect by saying "What do you think?" or "What would you expect?"
- Participant speaks after completing tasks: Encourage them to speak during the process
- Participant seeks approval: Reassure that there are no right or wrong answers

**Example Workshops Agenda**

| Health Catch-Up Implementation Workshop 1 | | | |
| --- | --- | --- | --- |
| Aims of the morning:   1. Explore what a successful screening intervention looks like from the migrant perspective 2. Discuss Health Catch-UP! and develop key messages to describe it to migrant groups using both effective language and visuals 3. Discuss alternative ways the tools can be delivered (e.g. at GP practice New Health Check, at Community Outreach events, when attending the GP for something else) and decide what patient support materials might be used to maximise understanding and engagement | | | |
| Please note this event is being held in multiple languages. Therefore information presented from the front needs to be delivered a sentence at a time, then allowing a pause to enable translation at each table. | | | |
| **Time** | **Action** | **Notes** | **By who?** |
| 09.00 | Arrive for set-up |  |  |
| 09.30– 10.15 | Coalition Meeting |  |  |
| Coffee available from 10.30 | | | |
| 10.45 – 10.50 | Welcome *(from front)* | Welcome to venue, logistical details, structure of morning, inspiration for the project |  |
| 10.50 – 11.00 | Introduction to Aims of the Day *(from front)* | - Define screening: having a test for something even though you aren’t feeling ill. - Give examples from ID/ NCD/ risk factors / cancer / mental health - Explain the concept of Health Catch-up as a tool to help primary care practitioners ensure migrants receive all the screening they are entitled to, especially on arrival to the UK |  |
| 11.00– 11.30 | Case Study Session  *(at tables)* | - Each participant to consider if they can/ are ok to answer: ‘Tell me about a time you have been screened’ (ideally in the UK) for approx. 5mins - Participants provided with *list of prompts/probes* (What was good about that? How did you feel? What could have been done better?) etc - Facilitator to document learning | Table based facilitators |
| 11.30 – 11.45 | Understanding Good Screening *(at tables)* | - Reflecting on the examples shared, group to design a flipchart page to answer the question: ‘What does good migrant health screening look like?’ - Facilitators provided with a *list of prompts/probes* about location, healthcare staff, actions / approach / what information, if different for some conditions | Table based facilitators |
| 11.45 – 12.00 | Sharing  *(from front)* | - Facilitator of each table presents results back to the group. | Facilitators |
| 12.00 – 12.15 |  | Coffee Break |  |
| 12.15 – 12.30 | Introduction to Health Catch-UP! *(from front)* | - More detail on information required for HCU - More detail on process of HCU - Mention of different HCU framing discussed so far - Explain need for framing (language & visual) |  |
| 12.30– 12.50 | Framing Health Catch-UP! *(at tables)* | - *Colour pens and paper at each table* - Participants encouraged to write key words / short phrases / intro sentences or images to frame HCU | Table based facilitators |
| 12.50 – 13.30 |  | Lunch |  |
| 13.30 – 14.10 | Health Catch-UP delivery models *(at tables)* | - *Delivery worksheets to discuss* at tables: GP New Patient Health Check, Outreach, GP opportunistic   What patient information resources would be needed to help understand HCU in each model? | Table based facilitators |
| 14.10– 14.30 | Summary | Gather together desired patient information resources and learning from the day. |  |

**Intervention planning table: Template**

| **Barrier/ *facilitator* to target behaviour**  Barriers shown in standard font  *Facilitators shown in italics*  EC = Expert consensus (PPI/stakeholder input)  L = Evidence from literature  Q = Qual research w users | **Detail about the evidence for barrier/ facilitator/ intervention ingredient** | **Possible intervention ingredient to promote facilitators and overcome barriers** | **Target construct and intervention function (COM-B, Behaviour Change wheel)** |
| --- | --- | --- | --- |
| **Target behaviour:** | | | |
|  |  |  |  |
|  |  |  |  |
|  |  |  |  |
|  |  |  |  |
|  |  |  |  |
|  |  |  |  |
|  |  |  |  |
|  |  |  |  |

**Example table of changes**

| ***[intervention name]*** | | | | | | |
| --- | --- | --- | --- | --- | --- | --- |
| ***Intervention element*** | ***Negative Comments*** | ***Positive Comments*** | ***Possible Change*** | ***Reason for change*** | ***Agreed change / NC*** | ***MoScoW*** |
|  |  |  |  |  |  |  |
|  |  |  |  |  |  |  |

**PBA coding framework**

| Coding framework | | |
| --- | --- | --- |
| Code | **Stands for** | **Means** |
| IMP | Important for behaviour change | This is an important change that is likely to impact behaviour change or a precursor to behaviour change (e.g. acceptability, feasibility, persuasiveness, motivation, engagement), and/or is in line with the Logic Model, and/or is in line with the Guiding Principles For example, participants appear unconvinced by an aspect of the intervention, so you decide to add motivational examples. |
| EAS | Easy and uncontroversial | An easy and feasible change that doesn’t involve any major design changes. For example, a participant was unsure of a technical term, so you add a definition. |
| REP | Repeatedly | This was said repeatedly, by more than one participant. |
| EXP | Experience | This is supported by experience. Please specify what kind of experience, for example:   1. PPIs agree this would be an appropriate change. 2. Other stakeholders (e.g. practitioners, providers, topic specialists) agree that this would be an appropriate change. 3. Literature: This is supported by evidence in the literature. |
| NCON | Does not contradict | This does not contradict experience (e.g. evidence), or the Logic Model, or the Guiding Principles |
| NC | Not changed | It was decided not to make this change. Please explain why (e.g. it would not be feasible; or only one person said this). |
