# Supplementary material for: How can we improve migrant health checks in UK primary care: ‘Health Catch-UP!’ a protocol for a participatory intervention development study
Source: BMJ Open. 2025 Nov 29;15(11):e106484. doi: 10.1136/bmjopen-2025-106484 (PMC12666209; doi:10.1136/bmjopen-2025-106484)
Supplement: Supplementary file 2 [file bmjopen-2025-106484.reviewer_comments.pdf]

PEER REVIEW HISTORY

BMJ Open publishes all reviews undertaken for accepted manuscripts. Reviewers are asked to complete a checklist review form and are provided with free text boxes to elaborate on their assessment. These free text comments are reproduced below.

ARTICLE DETAILS

Title (Provisional)

How can we improve migrant health checks in UK primary care: ‘Health Catch-UP!’ a protocol for a participatory intervention development study

Authors

Carter, Jessica; Knights, Felicity; Mackey, Kathryn; Deal, Anna; Hassan, Eltayeb; Trueba, Jose; Jayawardhena, Nimal; Alfred, Janet; Al-Sharabi, Isra; Ciftci, Yusuf; Aspray, Nathaniel; Harris, Philippa; Jayakumar, Subash; Seedat, Farah; Sanchez-Clemente, Nuria; Hall, Rebecca; Majeed, Azeem; Harris, Tess; Requena Méndez, Ana; Zenner, D; Tonkin-Crine, Sarah; Hargreaves, Sally

---

VERSION 1 - REVIEW

Reviewer 1

Name Lander, Jonas

Affiliation Hannover Medical School, Institute for Epidemiology, Social Medicine and Health Systems Research

Date 02-Jul-2025

COI None

Many thanks for inviting me to do a methodological review for this study protocol, and therefore I have indeed focused only on the methodological aspects. Please find my individual comments in the attached file, they should also include an indication of where improvement is needed more generally.

Development of an Implementation Package for the Health Catch-UP! Migrant Health Screening Tool for adults in Primary Care: Protocol for a Person-Based Approach Study

Comments

- Title: based on my own experiences with this, I would suggest to try shorten the title to no more than 15 words to increase visibility and readability
- Also, person-based approach study will rather not be clear to many readers in terms of what method this is…
- Abstract: you mention in the sentence on objective that this is a protocol, so how can it “evaluate this approach” at the same time?
- Abstract: any migrants? Can you be more specific?

- Abstract: what is “delivery models”?
- Abstract: you don’t mention where the focus groups belong in step 1-3
- “PBA and participatory process” > that sounds confusing with regard to where participation takes place, it reads like everything is p.
- Strengths and limitations: I believe bmjopen prefers these points to be shorter?
- Methods:
- Have you provided a description anywhere in the manuscript re what participation and any related term means to you (and how it is defined elsewhere?) > you certainly aware “participation” is an often over-used term that can refer to many things, e.g. focus groups and interviews aren’t participatory in that sense, which I also like you to check how you handle these terms throughout the manuscript
- Last sentence of context: please split in 2
- not sure if “study” aim should be kind of repeated here, you already mention in the intro?
- Seems like you don’t technically introduce the MHCRN abbreviation though I see what it refers to
- You “discussed” sth with 50 members, how did that work?
- The section “study planning”, Im not sure if this sth that can be in a study protocol, study planning could mean anything – please consider handling this in a different way?
- Same is basically true for the next section on study design. The methods start with “context”, so why another section on design here? It seems that this first part on context, aims, planning, design could really be sharpened…
- Do you introduce and define “PBA” somewhere early in the text?
- “the combination of qual methods with theory and evidence” > what is that?
- “the pba therefore helps integrate…” again I find this a complex sentence with too many info and concepts in it
- Pls try avoid abbreviations in figures etc
- Fig 2: planning and development could be perceived as being the same in terms of language?
- “PHCPS will be recruited through word of mouth” > how does that work?
- As already indicated above, pls specify e.g. what makes a workshop participatory and how is this more participatory then interviews? Participation so you get your data? Participation so migrants voice is heard in how you do the implementation later on? This is not clear but quite important?
- For instance, it would be really helpful to clarify how the logic model benefits from participation, how do you do this really? “With input from the coalition”, that could also just be an interview?
- Interv planning table to “bring together all the av evidence” > this also sounds quite complex, what if there are tons of evidence and how does brining together work in more technical / procedural terms?
- PHCP workshops “will all be highly interactive” > how can you know that in advance?
- So overall up until incl intervention development, you tend to be too complex, vague with several terms and too unspecific with explaining e.g. how you develop contents for the intervention/impl process more technically – it would be helpful to go through the important parts again and increase understanding on this

VERSION 1 - AUTHOR RESPONSE

**Reviewers Comments**	
Title: based on my own experiences with this, I would suggest to try shorten the title to no more than 15 words to increase visibility and readability	Thank you, we have shortened as suggested keeping in mind the above suggestions and requests of the editor.
Also, person-based approach study will rather not be clear to many readers in terms of what method this is…	Thank you, we have removed from the title and expanded in the text.
Abstract: you mention in the sentence on objective that this is a protocol, so how can it "evaluate this approach" at the same time?	This has been clarified in the abstract– we are evaluating our approach to participatory intervention development.
Abstract: any migrants? Can you be more specific?	We have included a definition in the abstract
Abstract: what is "delivery models"?	Definition included in the abstract
Abstract: you don't mention where the focus groups belong in step 1-3	Thank you we have rectified this included in step 2
"PBA and participatory process" > that sounds confusing with regard to where participation takes place, it reads like everything is p.	We have re-written this sentence so that it is clearer.
Strengths and limitations: I believe bmjopen prefers these points to be shorter?	We have revised these to be more specific and shorter.
Methods:	
Have you provided a description anywhere in the manuscript re what participation and any related term means to you (and how it is defined elsewhere?) > you certainly aware "participation" is an often over-used term that can refer to many things, e.g. focus groups and interviews aren't participatory in that sense, which I also like you to check how you handle these terms throughout the manuscript	Thank you this is an important point and we have reflected and clarified this through adding a definition box 2 and expanding on our explanations in the methods.
Last sentence of context: please split in 2	Sentence has been shortened.
not sure if "study" aim should be kind of repeated here, you already mention in the intro?	Thank you we have removed this and expanded slightly in the introduction
Seems like you don't technically introduce the MHCRN abbreviation though I see what it refers to	Thank you we have added this.
You "discussed" sth with 50 members, how did that work?	This was in the form of an ideas café which has been clarified in the text
The section "study planning", Im not sure if this sth that can be in a study protocol, study planning could mean anything – please consider handling this in a different way?	Thank you, we recognise this and have moved this section out of the methods and labelled as preparatory community engagement to better reflect the purpose.
Same is basically true for the next section on study design. The methods start with "context", so why another section on design here? It seems that this first part on context, aims, planning, design could really be sharpened…	Thank you for this observation, we have removed the aims section and integrated it into the introduction, we have removed the context section and combined this into a preparatory

	community engagement section and have also introduced the concept of the PBA into the introduction so that this section is sharper.
Do you introduce and define "PBA" somewhere early in the text?	This has been expanded upon in the introduction.
"the combination of qual methods with theory and evidence" > what is that?	We have simplified and clarified this section for readability.
"the pba therefore helps integrate…" again I find this a complex sentence with too many info and concepts in it	We have reflected on this and re-written this section of the methods with a shorter and sharpened explanation of the PBA.
Pls try avoid abbreviations in figures etc	Thank you we have expanded these
Fig 2: planning and development could be perceived as being the same in terms of language?	Thank you we acknowledge this, this is the format of the person-based approach, and a reference has been included and phases expanded upon above the figure.
"PHCPS will be recruited through word of mouth" > how does that work?	Thank you we have clarified this as snowball sampling from initial participants.
As already indicated above, pls specify e.g. what makes a workshop participatory and how is this more participatory then interviews? Participation so you get your data? Participation so migrants voice is heard in how you do the implementation later on? This is not clear but quite important?	Thank you, as outlined above we have included our definition of participatory research for clarification earlier in the manuscript. We have also expanded on the nature of the participatory workshops within the methods section of the text.
For instance, it would be really helpful to clarify how the logic model benefits from participation, how do you do this really? "With input from the coalition", that could also just be an interview?	Thank you we have provided explanation/examples of how we will approach this.
Interv planning table to "bring together all the av evidence" > this also sounds quite complex, what if there are tons of evidence and how does brining together work in more technical / procedural terms?	Thank you, we have expanded on our explanation of this and included an example template table of changes in the supplementary material for clarification.
PHCP workshops "will all be highly interactive" > how can you know that in advance?	We have changed this to workshops will be designed to be highly interactive and provided some examples of activities.
So overall up until incl intervention development, you tend to be too complex, vague with several terms and too unspecific with explaining e.g. how you develop contents for the intervention/impl process more technically – it would be helpful to go through the important parts again and increase understanding on this	Thank you, we have approached the manuscript with this feedback in mind and made the above outlined changes to simplify the methods section, add further explanations/specific examples where clarification was required and used the supplementary material to provide examples.

VERSION 2 - REVIEW

Reviewer 1

Name Lander, Jonas

Affiliation Hannover Medical School, Institute for Epidemiology, Social Medicine and Health Systems Research

Date 13-Oct-2025

COI

It seems that all of my previous comments have been addressed carefully in this revision and I therefore have no further points to add here.
